# The importance of local eosinophilia in the surgical outcome of chronic rhinosinusitis: a 3-year prospective observational study

**DOI:** 10.1186/2045-7022-3-S2-O12

**Published:** 2013-07-16

**Authors:** Stephan Vlaminck, Tom Vauterin, Bob Lerut

**Affiliations:** 1 AZ St John’s Hospital, ENT/Rhinology, Bruges, Belgium

## Background

Patients with chronic rhinosinusitis with/without nasal polyps (CRSwNP/CRSsNP) benefit from endoscopic sinus surgery (ESS), with an estimated success rate of 80%. At present, it remains unclear to what extent the presence of eosinophils, eosinophilic mucin (EM) and fungal hyphae (FH) in secretions influence the clinical outcome and recurrence of disease after ESS.

## Objective

By delineating CRS groups and subgroups based on the finding of eosinophils, EM and FH, differences in the frequency of recurrent disease after ESS over a longer period of time were investigated.

## Methods

A prospective mono-centre study including 221 CRS patients who were unresponsive to medical treatment and underwent ESS, was performed. All tissue and sinonasal secretions were microscopically examined for the presence of eosinophils, EM and FH. Patients were followed for 3 years after surgery. Recurrence was defined according to the EPOS clinical control assessment, based on nasal endoscopy, symptoms and the need for systemic treatment.

## Results

In total, 96 CRSwNP and 125 CRSsNP patients were included. Eosinophils were found in 78% of CRSwNP patients compared to 42% in CRSsNP. Eosinophilic mucin was observed in 52% of the CRSwNP group versus 20% of the CRSsNP group. Furthermore, secretion analysis revealed FH in 11% CRSwNP patients compared to 3% CRSsNP patients. Recurrence in the total group was 22% over 3 years. CRSwNP patients with eosinophilic involvement showed a recurrence rate of 48%. When the airway mucus secretions were positive for EM and FH the recurrence rate was even 73%.

## Conclusion

The presence of eosinophils greatly increases the risk of recurrent disease in CRSwNP patients. The finding of EM and FH in the collected sinonasal airway mucus secretions provides valuable information regarding the clinical outcome and the increased likelihood of CRS recurrence after ESS.

